# A mutant fitness assay identifies bacterial interactions in a model ocean hot spot

**DOI:** 10.1073/pnas.2217200120

**Published:** 2023-03-15

**Authors:** Jeremy E. Schreier, Christa B. Smith, Thomas R. Ioerger, Mary Ann Moran

**Affiliations:** ^a^Department of Marine Sciences, University of Georgia, Athens, GA 30602; ^b^Department of Computer Science and Engineering, Texas A&M University, College Station, TX 77843

**Keywords:** bacteria, phytoplankton, phycosphere, community ecology, Transposon Sequencing

## Abstract

Bacteria that assemble in phycospheres surrounding living phytoplankton cells metabolize a substantial proportion of ocean primary productivity. Yet the type and extent of interactions occurring among species that colonize these micron-scale “hot spot” environments are challenging to study. We identified genes that mediate bacterial interactions in phycosphere communities by culturing a transposon mutant library of copiotrophic bacterium *Ruegeria pomeroyi* DSS-3 with the diatom *Thalassiosira pseudonana* CCMP1335 as the sole source of organic matter in the presence or absence of other heterotrophic bacterial species. The function of genes having significant effects on *R. pomeroyi* fitness indicated explicit cell–cell interactions initiated in the multibacterial phycospheres. We found that *R. pomeroyi* simultaneously competed for shared substrates while increasing reliance on substrates that did not support the other species’ growth. Fitness outcomes also indicated that the bacterium competed for nitrogen in the forms of ammonium and amino acids; obtained purines, pyrimidines, and cofactors via crossfeeding; both initiated and defended antagonistic interactions; and sensed an environment with altered oxygen and superoxide levels. The large genomes characteristic of copiotrophic marine bacteria are hypothesized to enable responses to dynamic ecological challenges occurring at the scale of microns. Here, we discover >200 nonessential genes implicated in the management of fitness costs and benefits of membership in a globally significant bacterial community.

## Significance

Ecological interactions that occur between phycosphere-associated bacteria at the micron scale have the potential to influence a major fraction of annual carbon flux at the global scale. Despite the importance of microbial carbon flow, studying the ecology of these microenvironments remains challenging. Fitness measures of bacterial transposon mutants identified four primary classes of interactions among members of a model bacterial community provisioned by a cocultured diatom. This work advances our understanding of ecological associations in multispecies microbial environments.

Of the ~63 Pg of atmospheric carbon converted into surface ocean organic matter by marine phytoplankton each year (1), approximately 20 Pg are released as metabolites from living cells (2, 3). These labile components of the dissolved organic carbon pool are taken up by heterotrophic marine bacteria, initiating a process that results in a large and rapid flux between phytoplankton productivity and global carbon reservoirs (4, 5). The mechanisms governing this flux of dissolved primary production through heterotrophic microbes are still poorly understood, stemming from challenges of studying the ecology of micron-scale environments.

Organic compounds released by living phytoplankton accumulate in a region around the cell referred to as the phycosphere (6), one type of microbial hot spot (4) where bacteria are concentrated to take advantage of higher carbon and nutrient availability (7). Phycosphere organic matter is diverse in its composition, characterized by compound classes that include carbohydrates, amino acids, osmolytes, organic sulfur, nucleobases, and signaling molecules, among others (8–10). Consequently, a diversity of bacteria able to benefit from phytoplankton metabolites colonize phycospheres, typically including members of the Alphaproteobacteria (frequently the *Rhodobacteraceae*), Gammaproteobacteria, and Bacteroidetes (11–13). The bacterial species colonizing phycospheres most likely compete for metabolites (14–16), inorganic nutrients, essential metals, and other resources (17, 18). Such competitive interactions have been proposed to explain the dominance in phycosphere communities of copiotrophic bacteria, those having large, well-regulated genomes and capacity for rapid growth when conditions are favorable (19).

**Fig. 1.**
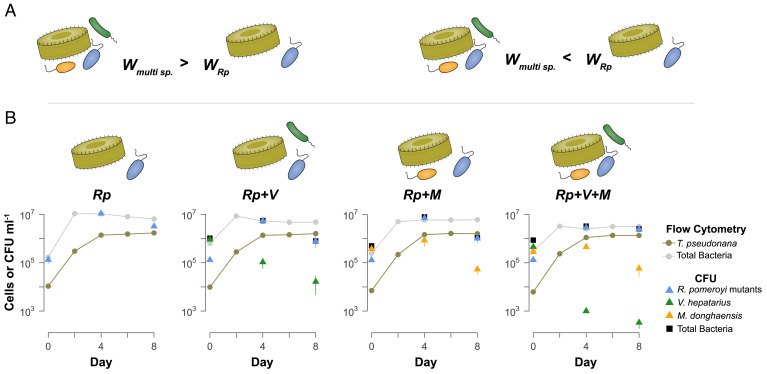
Overview of the model phycosphere experiment. (*A*) Fitness of *R. pomeroyi* mutants in multibacterial communities (*Rp*+*V*; *Rp*+*M*; *Rp*+*V*+*M*) was compared to fitness in single-bacterial cultures (*Rp*). Higher fitness (*W*_multi sp._> *W_Rp_*) indicated that the missing function was rescued or superfluous when growing with other species, for example through crossfeeding. Lower fitness (*W_multi sp._< W_Rp_*) indicated that the missing function would have been beneficial when growing with other species, for example, by increasing competitiveness. (*B*) Growth of community members over 8 d. SEM (n = 4) is displayed for all points; some fall within the symbols. Solid lines indicate diatom and bacterial density based on flow cytometric analysis (cells mL^−1^). Triangles indicate abundance of individual bacterial species, and squares represent abundance of all bacteria (CFU mL^−1^) at three time points.

While ecological associations among phycosphere bacteria have the potential to influence rates and efficiencies of carbon flux at the global scale, the interactions themselves occur at the micron scale (20). Here, we detect interactions occurring among phycosphere bacteria through the identification of genes influencing fitness. In a model phycosphere containing diatom *Thalassiosira pseudonana* CCMP1335 as the phytoplankton host, *Rhodobacteraceae* bacterium *Ruegeria pomeroyi* DSS-3 was inoculated in the form of a random transposon mutant pool either alone or sharing the coculture with one or both of two other bacteria, *Vibrio hepatarius* HF70 and *Marivivens donghaensis* HF1. The bacteria selected for this study were the three most abundant members of a coastal community enriched on diatom metabolites (14, 21). In the case of *R. pomeroyi*, the bacterium had also been isolated previously from the same coastal location where the enrichment culture inoculum was obtained (14, 22) and is amenable to genetic manipulation (23, 24). In this study, *R. pomeroyi* mutant populations recovered from the model system after 8 d were analyzed by Transposon Sequencing (TnSeq) (25–27) to determine fitness effects of each single-gene knockout in the presence and absence of other bacteria. Those with lower growth rates in multibacterial communities were regarded as having disruptions in genes that would have been beneficial in coculture with other species if remaining functional. Those with higher growth rates in multibacterial communities were regarded as having disruptions in genes that would have been unnecessary or costly in the presence of other species if remaining functional (Fig. 1*A*). Differential growth rates among the mutant pool (~60,000 distinct mutants) provided insights into potential mechanisms of interspecies interactions in a model marine hot spot of biogeochemical importance.

## Results and Discussion

We established four model bacterial communities reliant on the extracellular release of photosynthate continually generated by cocultured diatom *T. pseudonana* in a medium replete with phosphate, nitrate, vitamins B_12_, B_7_, B_1_, and trace metals. The *R. pomeroyi* transposon mutant pool was inoculated into the diatom cultures as the sole bacterial species (treatment referred to as *Rp*), or along with *V. hepatarius* (*Rp+V),* or *M. donghaensis* (*Rp+M*), or both (*Rp+V+M*) (Fig. 1*B* and *SI Appendix*, Fig. S1). The same number of *R. pomeroyi* cells was inoculated into all model communities to ensure equal mutant diversity, and therefore the total bacterial density in multibacterial communities was initially 1.4- to 3.7-fold greater than that in the single-bacterium treatment. This difference, however, was not reflected in the mature cultures, likely representing the carrying capacity set by substrate release from the living diatom (Fig. 1*B*). The *R. pomeroyi* mutant pools experienced an average of 5.5 generations during the 8-d experiment as determined by colony-forming units (CFUs). CFUs of *V. hepatarius* declined by ~2 to 4 orders of magnitude, with a more pronounced decline when both *R. pomeroyi* and *M. donghaensis* were present (*Rp+V+M*). *M. donghaensis* increased 2-fold and then gradually declined (Fig. 1*B*). Diatom growth (Fig. 1*B*) and photosynthetic physiology (F_v_/F_m_; *SI Appendix*, Fig. S2) were indistinguishable across treatments.

### Mutant Fitness Overview.

**Fig. 2.**
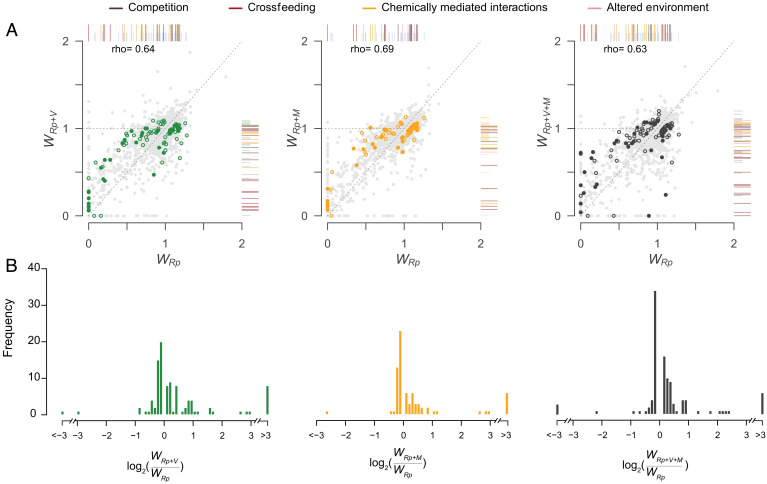
Effect of multibacterial communities on *R. pomeroyi* mutant fitness. (*A*) Correlation between mutant fitness (*W*) in multi- (*y* axis) versus single- (*x *axis) bacterial treatments (Spearman’s rho, *P* < 0.05 for all). Dashed lines indicate a 1:1 relationship. Mutants cover 86% of protein encoding genes, with most genes missing from the mutant pool-mediating functions essential for cell viability. Symbols with color indicate significantly different fitness for mutants in multibacterial treatments versus the single-bacterial treatment based on Benjamini–Hochberg adjusted *P* values from randomization tests of the log_2_ fold-change of fitness (*P-adj* < 0.05). Colored symbols that are also filled are included in Tables 1–4. Rug plots on the right and top of plots show the distribution of *W* for mutants with significantly different fitness; longer rug markers are colored to represent the four ecological modes of interaction. Colored points close to the 1:1 line have small but statistically significant fitness differences. (*B*) Distribution of the log_2_ fold-change of fitness for multibacteria treatments compared to the single-bacterial (*Rp*) treatment. Positive values indicate mutant fitness increases in multispecies communities; negative values indicate fitness decreases. Mutants with fitness changes greater than |3| were binned.

Fitness was calculated for the 3,748 *R. pomeroyi* genes having at least one curated transposon insertion site (defined as insertions within the central 90% of a coding region); these represented 86% of the 4,293 total coding regions. To calculate fitness (*W*), a mutant’s change in abundance over 8 d was compared to that of the combined mutant pool (26). Thus, a mutant with a fitness of one had a growth rate equivalent to the combined mutant pool in the same treatment, whereas a mutant with a fitness of 0.5 was growing at half the average rate of all mutants. Although the fitness of *R. pomeroyi* mutants was generally correlated between the single- and multi-bacteria cultures (Fig. 2*A*), those with significant differences revealed the specific genes for which loss of function affected *R. pomeroyi*’s ability to interact with the bacteria sharing the phycosphere. The magnitude of the treatment effect was represented by log_2_ fold-change of *R. pomeroyi* mutant fitness in multibacterial communities relative to single-bacterium treatments (Fig. 2*B*).

**Fig. 3.**
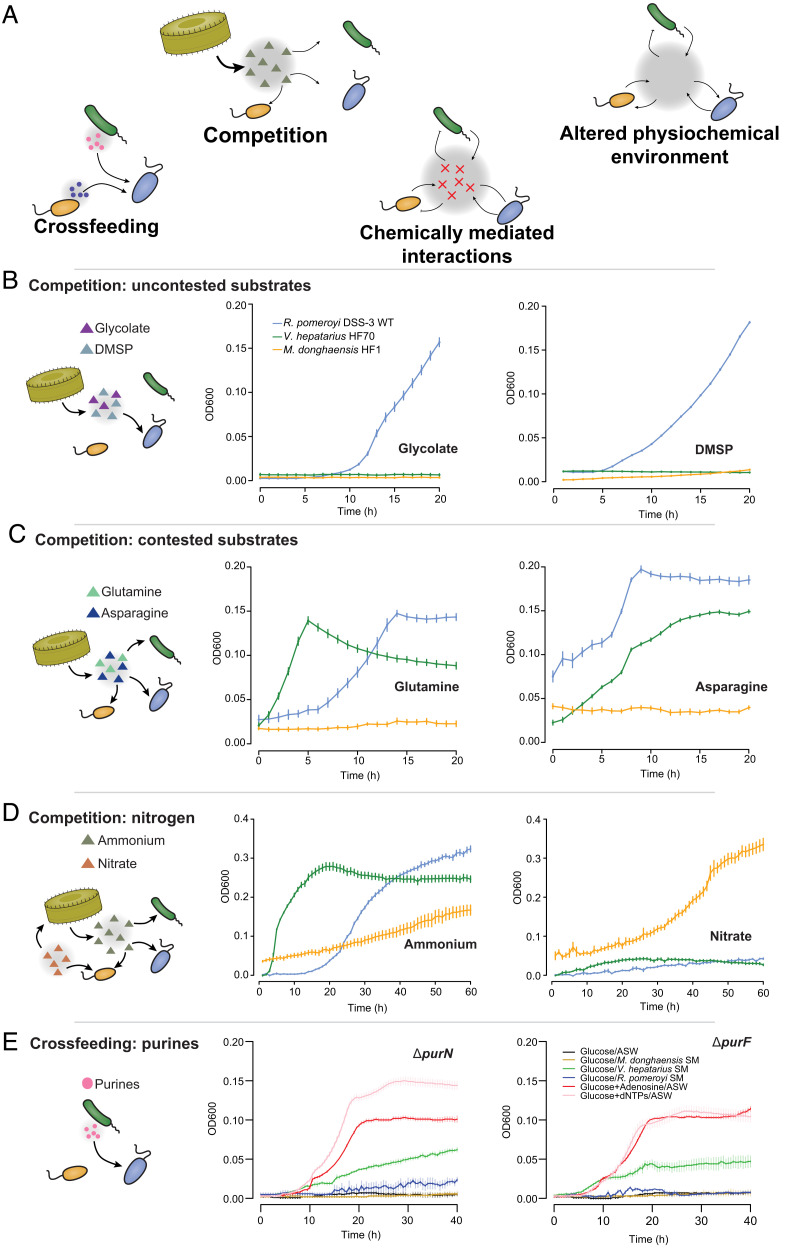
Experimental tests of bacterial interaction hypotheses. (*A*) Four categories of bacterial interactions implicated by phycosphere mutant fitness assays. (*B*) Minimal medium with glycolate or DMSP (12 mM carbon) as the sole carbon source supported strong growth only of *R. pomeroyi,* suggestive of increased importance of a noncontested resource. (*C*) Minimal medium with glutamine or asparagine (12 mM carbon) as the sole carbon source supported growth of both *V. hepatarius* and *R. pomeroyi,* suggestive of a communal carbon source. (*D*) Minimal glucose medium with ammonium or nitrate as the inorganic nitrogen source. Only *M. donghaensis* can assimilate nitrate, consistent with exploitative competition for ammonium observed only between *R. pomeroyi* and *V. hepatarius*. (*E*) Growth of isolated transposon mutants *purN* and *purF* in minimal glucose medium amended with either spent medium, adenosine, or dNTPs. Mutant auxotrophy is confirmed by lack of growth in glucose alone. Rescue of auxotrophy is demonstrated by addition of adenosine, dNTPs, or *Vibrio* spent medium, supporting the hypothesis of purine crossfeeding.

When mutants having significant differences in fitness were analyzed based on function of the deleted gene and direction of fitness change, four phenomenological modes of ecological interactions emerged from the data: i) vying for resources via competition (13% of significant mutants), ii) obtaining resources via crossfeeding (11%), iii) invoking chemically mediated interactions via secondary metabolites (4%), and iv) adapting to altered environmental conditions (10%) (Fig. 3*A*)**.** The remaining mutants had genes disrupted in central metabolism (12%), regulation (6%), mixed functions not well annotated or not clearly linked to bacterial interactions (25%), and hypothetical proteins (19%) (Dataset S1).

### Competition.

Twenty-six mutants with significant fitness differences had nonfunctional genes annotated for resource acquisition, encompassing several different arenas of bacterial competition in the *T. pseudonana* phycosphere. One of these was competition for substrates, as evidenced by fitness differences in 22 mutants disrupted in the transport or catabolism of organic molecules (Table 1). Most substrate mutants had lower fitness in multibacterial treatments (plotting below the 1:1 line in Fig. 2*A*); they included mutants for transport of dicarboxylic acid-like molecules, and for catabolism of glycolate, dimethylsulfoniopropionate (DMSP), carnitine, taurine, threonine, and methylamine. The remaining five substrate mutants instead had higher fitness in multibacterial treatments (plotting above the 1:1 line in Fig. 2*A*); these included mutants for transport of sugars and a nitrogen-rich amino acid (glutamine, glutamate, aspartate, and/or asparagine).

**Table 1.** Fitness differences (log_2_-fold) of selected mutants involved in competition whose growth was significantly influenced in the multibacterial treatments

Gene	Protein name (Gene symbol)	Function	Rp+V	Rp+M	Rp+V+M
SPO3479	glycolate oxidase (*glcE)*	Carbon source – Glycolate	−0.44		
SPO3805	Methylthioacryloyl-CoA hydratase (*dmdD*)	Carbon source – DMSP		−0.11	
SPO2708	carnitine dehydratase	Carbon/nitrogen source – carnitine	−0.16		
SPO1586	N-methylglutamate dehydrogenase (*mgdC*)	Carbon/nitrogen source – methylamine		−0.18	
SPO3360	2-amino-3-ketobutyrate co-A ligase (*kbl*)	Carbon/nitrogen source – threonine			−0.12
SPO0673	taurine--pyruvate aminotransferase (*tpa*)	Carbon/nitrogen source – taurine		−0.2	
SPO0397	protein-P-II uridylyltransferase (*glnD*)	Nitrogen acquisition			−6.5
SPO3661	allophanate hydrolase family protein	Nitrogen acquisition	−0.24	−0.24	−0.29
SPO0891	alkylphosphonate utilization protein (*phnM*)	Phosphate acquisition		−0.11	
SPO1727	polyphosphate kinase 2, putative	Phosphate storage			−2.2
SPO1213	oligopeptide ABC transporter, ATP-binding	Transporter – peptide		−0.12	
SPO2815	peptide/nickel/opine ABC transporter, permease	Transporter	−0.12		
SPO2816	peptide/nickel/opine ABC transporter, permease	Transporter	−0.14		
SPO1814	TRAP dicarboxylate transporter (*dctP*)	Transporter – organic acid	−0.21	−0.2	−0.18
SPO1816	TRAP dicarboxylate transporter (*dctM*)	Transporter – organic acid		−0.17	−0.18
SPO2626	TRAP transporter (*dctM*)	Transporter – organic acid	−0.13		−0.12
SPO2627	C4 dicarbodylateTRAP transporter (*dctQ*)	Transporter – organic acid	−0.17		−0.65
SPO2630	C4-dicarboxylate TRAP regulatory protein	Transporter – organic acid	−0.25		
SPOA0238	TRAP dicarboxylate transporter (*dctP*)	Transporter – organic acid	−0.18	−0.2	−0.18
SPOA0240	TRAP transporter (*dctM*)	Transporter – organic acid	−0.14	−0.14	−0.14
SPOA0237	C4-dicarboxylate transport transcriptional regulatory protein (*dctD*-2)	Transporter – organic acid	−0.27	−0.25	−0.22
SPO0521	glutamate/glutamine/aspartate/asparagine ABC transporter, permease	Transporter – N-rich amino acid			0.22
SPO0715	phosphocarrier protein HPr	Transporter – sugar		1.2	
SPOA0249	TRAP dicarboxylate transporter (*dctP*)	Transporter – organic acid			0.13
SPO1496	ABC transporter, permease	Transporter		0.38	
SPOA0367	ABC transporter, permease	Transporter	0.12		

Empty cells represent fitness changes that were not significantly different based on randomization tests.

We considered a possible explanation for these two opposite fitness outcomes, hypothesizing that when substrates of disrupted genes are uncontested resources, available only to *R. pomeroyi*, they will be of increased importance for fitness in multibacterial communities and lead to lower fitness relative to the combined mutant pool. On the contrary, when substrates are communal resources and *R. pomeroyi* is less successful in attaining them when other bacteria are present, they will be of decreased importance to fitness in multibacterial communities and lead to higher relative fitness. Predictions following from this hypothesis are that in the first case, the substrates of the transporter/catabolic genes support growth of *R. pomeroyi* only, while in the second, the substrates support growth of *R. pomeroyi* plus at least one other species.

This hypothesis was tested by inoculating wild-type *R. pomeroyi*, *V. hepatarius*, and *M. donghaensis* individually into media containing one of the six substrates selected based on well-annotated genes with known target molecules in the *R. pomeroyi* genome (Table 1). All organic compounds supported growth of *R. pomeroyi* (Fig. 3*B*). Glycolate and DMSP, whose catabolism mutants had lower fitness in multibacterial communities, indeed supported growth only of *R. pomeroyi*, consistent with roles as uncontested resources. However, glutamine, asparagine (Fig. 3*C*), aspartate, and glutamate (*SI Appendix*, Fig. S3), whose transporter mutants had increased fitness in multibacterial communities, supported the growth of *R. pomeroyi* and at least one other species, consistent with roles as communal resources. This bacterium’s fitness in the phycosphere thus appeared affected by its abilities to both compete for substrates that are shared, and have access to those that are less commonplace. *R. pomeroyi* devotes 12% of its genome to transporters, a high proportion compared to other Alphaproteobacteria (28); among these transporters are ~126 annotated for uptake of organic compounds.

A second group of mutants revealed a contest for phycosphere nutrients. First, *R. pomeroyi glnD* mutants had lower fitness phenotypes when in multibacterial communities. Bacteria with a disrupted *glnD* receive an artificial signal of nitrogen sufficiency (29), causing the downregulation of high-affinity ammonium assimilation; the fitness decreases were therefore interpreted as evidence for stronger ammonium limitation in multibacterial communities. Growth of *R. pomeroyi*’s *glnD* mutant was especially affected when *V. hepatarius* was a member of the community (>threefold greater fitness change compared to *Rp+M*) (Table 1 and Dataset S1). Genomic analysis revealed that all the three strains harbor genes to assimilate ammonium (which was not a component of the medium, and thus was available in the cultures only via regeneration). However, assimilation of nitrate (provided in excess in the culture medium) was possible only for *M. donghaensis* (*SI Appendix*, Table S1), decreasing its reliance on ammonium. These nitrogen uptake phenotype predictions were experimentally confirmed in growth assays with either ammonium or nitrate as the nitrogen source (Fig. 3*D*). A second *R. pomeroyi* nitrogen mutant was disrupted in an allophanate hydrolase gene, making it unable to release ammonium from urea. The decreased fitness of this mutant is consistent with stronger nitrogen limitation in the multibacterial communities. Nitrate was used as the nitrogen source for these model systems because it fuels most natural diatom blooms (30) and differences in genetic capabilities for nitrogen acquisition are known to affect marine bacterial success (31, 32).

Competition for phosphorus was indicated by two mutants in phosphate acquisition and utilization. Despite the phosphate-replete medium, fitness decrease of a phosphonate utilization mutant (*phnM*) suggested an increased reliance on dissolved organic phosphate. Genomic analysis indicated that *R. pomeroyi* is the only bacterium in the model phycosphere communities with a broad-specificity C-P lyase, suggesting another niche dimension that could decrease direct competition (33) (*SI Appendix*, Table S1). Fitness decreases in multispecies communities were observed for mutants of polyphosphate kinase (*ppk*) which are unable to utilize stored polyphosphate, an energetically economical source of phosphorous for ATP (34). Considering the total suite of 26 competition mutants, managing options for carbon, nitrogen, and phosphorous procurement was key to the competitive success of phycosphere bacteria, most likely outweighing the associated energetic costs of regulation (4, 20, 35).

### Crossfeeding.

Twenty-four mutants with significant fitness differences had nonfunctional genes in anabolic pathways (Table 2). All had higher fitness in multibacterial treatments (above the 1:1 line in Fig. 2*A*) with an average increase of 2.8-fold over the single-bacterial treatment (Dataset S1). This category included mutants with disruptions in the biosynthesis of purines (four genes), pyrimidines (two genes), essential amino acids (14 genes), vitamins B_5_ and B_12_ (two genes), and molybdopterin (two genes) (Table 2). The latter two categories represent metabolic cofactors that are energetically expensive to synthesize yet known to be released exogenously by some members of microbial communities, referred to as “public goods” (36–38). Mutant rescue could have resulted from metabolite export from the other species, as reported previously for a marine *Vibrio* species (39), or from release by stressed or dying cells (40, 41) during *V. hepatarius* or *M. donghaensis* cell density decreases (Fig. 1*B*). In either case, fitness increases indicated resource release at levels sufficient to rescue auxotrophs.

**Table 2.** Fitness differences (log_2_-fold) of selected mutants involved in crossfeeding whose growth was significantly influenced in the multibacterial treatments

Gene	Protein name (Gene symbol)	Function	Rp+V	Rp+M	Rp+V+M
SPO0018	argininosuccinate synthase (*argG*)	Arginine	3.5	3	
SPO0332	argininosuccinate lyase (*argH*)	Arginine	2.8		
SPO0422	2-isopropylmalate synthase (*leuA*)	Leucine		0.6	
SPO0210	3-isopropylmalate dehydrogenase (*leuB*)	Leucine			2.4
SPO0215	3-isopropylmalate dehydratase (*leuD*-1)	Leucine	1.1		0.77
SPO1351	O-succinylhomoserine sulfhydrylase (*metZ*)	Homocysteine	3		
SPO1734	homoserine dehydrogenase (*hom*)	Homoserine		5	
SPO1884	S-methyltransferase component of split *metH*	Methionine			3.9
SPO1973	3-dehydroquinate dehydratase, type II (*aroQ*)	Aromatic amino acids	3.9		
SPO2150	anthranilate phosphoribosyltransferase (*trpD*)	Tryptophan	3.6		2.3
SPO2151	indole-3-glycerol phosphate synthase (*trpC*)	Tryptophan	4.4		
SPO3768	glutamate synthase (*gltB*)	Glutamate	0.9		0.75
SPO2634	sulfite reductase	Amino acid – sulfur assimilation	4.8		
SPO2635	phosophoadenylyl-sulfate reductase (*cysH*)	Amino acid – sulfur assimilation		4.2	
SPO0102	3-methyl-2-oxobutanoate hydroxymethyltransferase (*panB*)	Vitamin B5	0.43	0.42	0.44
SPO3224	cobalamin biosynthetic protein (*cobC*)	Vitamin B12			6.2
SPO3633	molybdopterin converting factor, subunit 2	Molybdopterin	0.41		0.3
SPO3634	molybdopterin converting factor, subunit 1 (*moaD*)	Molybdopterin			0.3
SPO0284	dihydroorotase, multifunctional complex	Pyrimidine			2.2
SPO2654	orotate phosphoribosyltransferase (*pyrE*)	Pyrimidine	4.9		
SPO1318	adenylosuccinate synthetase (*purA*)	Purine		3.6	5.2
SPO1870	phosphoribosylformylglycinamidine synthase II (*purL*)	Purine			3.1
SPO2168	phosphoribosylglycinamide formyltransferase (*purN)*	Purine	1.6		
SPO2677	amidophosphoribosyltransferase (*purF*)	Purine	1.2		

Empty cells represent fitness changes that were not significantly different based on randomization tests.

This hypothesis of crossfeeding was tested by inoculating *R. pomeroyi* transposon mutants disrupted in purine biosynthesis gene (*purF, purN*) into spent medium from all the three species. Medium from *V. hepatarius* cultures rescued growth of the purine mutants, while medium from *M. donghaensis* and *R. pomeroyi* cultures did not, consistent with the interpretation of crossfeeding dominated by metabolites from *Vibrio* (Table 2 and Fig. 3*E*). Nucleotide crossfeeding among bacterial species has been found previously; for example, *Escherichia coli* purine auxotrophs were recused when cocultured with *Rhodopseudomonas palustris* (42) and dissolved free nucleotides have been documented in natural seawater and shown to be readily assimilated by bacterial communities (43). Metabolite exchange between coisolated strains (44) and predictions from genome-scale metabolic models (45–47) suggest that crossfeeding is a common feature of microbial communities. Here, fitness outcomes revealed metabolite exchange between phycosphere bacteria that at a minimum includes amino acids, nucleobases, and cofactors.

### Chemically Mediated Interactions.

Fitness changes in mutants that were disrupted in the synthesis or efflux of secondary metabolites pointed to potential bacterial interactions via ecological signaling molecules (5). The nine *R. pomeroyi* mutants in this category had disruptions in secretion and efflux systems, and in the synthesis of exogenous signaling compounds (Table 3), and these experienced either increases or decreases in fitness. For example, increased fitness of a type I secretion system mutant suggested that *R. pomeroyi* benefitted from avoiding the cost of toxin production or export, potentially because the excreted antimicrobial was ineffective against *V. hepatarius* and *M. donghaensis* or the other populations of *R. pomeroyi* mutants synthesized the toxin and provided protection. Decreased fitness of a type II secretion system (Table 3) suggested that *R. pomeroyi* was disadvantaged if unable to export accumulated toxins. Observations that the decline in *V. hepatarius* and *M. donghaensis* populations in the multibacterial communities (Fig. 1*B*) did not occur when the bacteria were grown in single-species culture with the diatom (*SI Appendix*, Fig. S2) support the hypothesized antagonism via antimicrobials. Further, agar plate assays demonstrated that *V. hepatarius* has the potential to inhibit the growth of *R. pomeroyi* (*SI Appendix*, Fig. S4), consistent with antimicrobial activity being a component of phycosphere ecology.

**Table 3.** Fitness differences (log_2_-fold) of selected mutants involved in chemically mediated interactions whose growth was significantly influenced in the multibacterial treatments

Gene	Protein name (Gene symbol)	Function	Rp+V	Rp+M	Rp+V+M
SPO1240	type I secretion outer membrane protein (*tolC*)	Antimicrobial/Efflux	0.9		0.9
SPO1928	Tat (twin-arginine translocation) pathway signal sequence domain protein	Antimicrobial/Efflux		−0.13	
SPO3091	type II secretion system protein F (*gspF*)	Antimicrobial/Efflux		−0.11	-0.13
SPO1757	capsular polysaccharide export protein (*kpsS*)	Efflux			-0.18
SPO2713	Protein translocase subunit SecA 2 (*secA*2)	Efflux		−0.42	
SPO2251	gene transfer agent (*orfg*14)	Gene transfer	−0.84		
SPO0071	competence protein F, putative	Gene transfer	−0.13		−0.2
SPOA0111	indolepyruvate oxidoreductase (*iorA*)	Signaling – auxin			−0.12
SPO2287	autoinducer synthesis protein	Signaling			0.12

Empty cells represent fitness changes that were not significantly different based on randomization tests.

### Altered Environment.

Mutants in this category had disruptions in genes that respond, directly or indirectly, to environmental conditions external to the cell that are hypothesized to be changed by the presence of other bacteria; mutants experienced either increases or decreases in fitness. Mutations in genes synthesizing the aa3-type cytochrome oxidase of the aerobic respiratory electron transport chain (four genes; Table 4) and nitric oxide reductase resulted in higher fitness, while those encoding alternative cytochromes such as c-554, P450, and the nitrite reductase cd1 resulted in lower fitness (three genes). Collectively, these suggest intensified daily dynamics in oxygen availability in the multispecies phycospheres to the point where bacterial fitness is impacted. Because our experimental systems were established with cell density and metabolite release rates comparable to those in surface seawater, these results should be relevant for natural phycospheres. Ephemeral oxygen depletion has been reported for marine particles such as microbial aggregates and fecal pellets (48), including photosynthetically active microenvironments (49, 50). Indeed, a flexible respiratory system to adjust to dynamic oxygen fluctuations has been proposed as a key trait for bacteria associating with marine phytoplankton (19, 51, 52), with which fitness outcomes of *R. pomeroyi* mutants concur.

**Table 4.** Fitness differences (log_2_-fold) of selected mutants indicative of an altered environment whose growth was significantly influenced in the multibacterial treatments

Gene	Protein name (Gene symbol)	Function	Rp+V	Rp+M	Rp+V+M
SPO3073	cytochrome c oxidase, subunit III (*ctaE*)	Aerobic respiration	0.43	0.33	
SPO3075	Protoheme IX farnesyltransferase (*ctaB*)	Aerobic respiration	0.87	0.68	0.86
SPO3076	cytochrome c oxidase, subunit II (*ctaC*)	Aerobic respiration		0.47	0.49
SPO1383	cytochrome c oxidase, aa3-type, subunit I (*ctaD*)	Aerobic respiration	0.43		
SPOA0212	nitric oxide reductase F protein	Respiration			0.31
SPO2099	cytochrome c-554 (*cycF*)	Respiration	−0.15		
SPO1898	cytochrome P450 family protein	Respiration	−0.14		
SPOA0223	cytochrome cd1 nitrite reductase (*nirF*)	Respiration	−0.17		
SPO0935	nitroreductase family protein	Respiration			−0.17
SPO2340	superoxide dismutase, Fe (*sodB*)	Reactive oxygen species	1.7		2.1
SPO_Sp16SA	16S rRNA	Cell cycle/growth			0.11
SPO_Sp16SB	16S rRNA	Cell cycle/growth			0.13
SPO_Sp16SC	16S rRNA	Cell cycle/growth			0.14
SPO0503	ribosomal protein L30 (*rpmD)*	Cell cycle/growth			0.34
SPO1443	ATP-dependent RNA helicase RhlE (*rhlE*)	Cell cycle/growth	0.44	0.36	0.42
SPO3256	ribosomal protein L31 (*rpmE*)	Cell cycle/growth		3.9	
SPO0691	GTP-binding protein Era (*era*)	Cell cycle/growth	0.78	0.84	
SPO2819	NAD(P)+ transhydrogenase, beta (*pntB*)	Energy	0.39	0.36	
SPO2820	NAD(P) transhydrogenase, alpha (*pntA*)	Energy			0.36
SPO0095	nicotinate phosphoribosyltransferase (*pncB*)	Energy	0.11		
SPO1519	carbon monoxide dehydrogenase, large subunit (*coxL*-1)	Energy – carbon monoxide	−0.17		−0.23
SPO3901	carbon monoxide dehydrogenase G protein, putative	Energy – carbon monoxide			−0.31

Empty cells represent fitness changes that were not significantly different based on randomization tests.

Higher fitness of mutants unable to synthesize superoxide dismutase (*sodB*) pointed to a reduced need to protect against this reactive oxygen species when sharing the phycosphere (Table 4). Superoxide is involved in cellular stress, signaling, and redox cycling of metals (53). While both *T*. *pseudonana* (54) and *R. pomeroyi* (55) release superoxide extracellularly, net production by *T. pseudonana* is three orders of magnitude greater than *R. pomeroyi* (54, 55) and other marine bacteria (56), and phytoplankton-produced superoxide can reach levels inhibitory to bacteria (57). The *sodB* mutant rescue is thus interpreted here as an indication of public goods provided by bacteria that scavenge superoxide radicals in the mixed-species communities.

In what may be a general response to the challenges of multispecies environments, *R. pomeroyi* mutants with disrupted rRNA genes, ribosomal protein genes, and regulatory genes for transcription and translation had higher fitness in multibacterial communities (seven genes). Accordingly, we hypothesize that maximally functioning protein translation machinery is less beneficial when other bacteria share the phycosphere. Bacteria respond to changes in external resource availability by altering their proteome partitioning (58, 59), and under conditions in which nutrient or substrate availability limits growth, decreased need for ribosome-related proteins has been observed (60). Consistent with this, *R. pomeroyi* had fewer generations in multibacterial communities (e.g., 4.4 in the *Rp*+*V*+*M* treatment compared to 6.4 in the *Rp* treatment; *SI Appendix*, Table S2). Mutants in *pntAB*, which mediates conversion of NADH to NADPH (61) (Table 4), also exhibited higher fitness in multibacterial communities, consistent with presumed lower demand for this anabolic energy molecule.

Similar to strategies of mycobacteria enduring hypoxia and slowed growth under carbon starvation, decreased fitness of two *R. pomeroyi* carbon monoxide (CO) dehydrogenase mutants suggested a potential reliance on alternative electron donors to remain energized(62). CO oxidation does not provide enough energy to support growth or alter intracellular metabolite pools in *R. pomeroyi* (63). Nonetheless, increased synthesis of CO enzymes has been observed under carbon starvation (64) and would allow the bacterium to exploit CO for cellular maintenance (65). Slowed growth of *R. pomeroyi* as other bacterial species assemble into the phycosphere may increase the fitness benefits of a functioning CO oxidation pathway.

## Conclusions

Direct fitness measures of single-gene mutants in a model ocean hot spot revealed how growth and activity of a focal bacterium were affected by community members. Apportionment of the available labile organic matter was governed by two factors: the bacterium’s ability to compete for communal substrates, and its capacity to utilize those less commonplace. The latter has been proposed to lay the groundwork for evolution of mutualistic relationships between marine phytoplankton and bacterial lineages (35, 66). Crossfeeding in bacterial communities is controversial regarding its extent and ecological relevance (44, 67). Here, *R. pomeroyi* fitness benefitted from ~13 central metabolites (amino acids, purines, pyrimidines, and cofactors) available in the multispecies communities in amounts sufficient to rescue auxotrophs. The general importance of metabolites produced by species sharing a phycosphere, regardless of the mechanism by which they are released, was underscored by 1.7-fold higher fitness gains among mutants disrupted in anabolic genes relative to those disrupted in catabolic genes or transporters (*SI Appendix*, Fig. S5). Nonetheless, the *R. pomeroyi* mutant pool achieved 30% fewer generations when other species shared the phycosphere, indicating an overall growth cost to life in a community.

Identification of authentic bacteria–bacteria interactions is challenging when experimental systems have substrate concentrations and bacterial growth rates considerably higher than those in natural ecosystems. By provisioning our model systems with exometabolites from cocultured diatom cells, we achieved an environment with high fidelity to a phycosphere hot spot in terms of substrate composition, substrate supply rate, microbial density, and spatial organization. In support of this, diatom and bacterial cell abundances in the model systems were consistent with those in natural diatom blooms (15, 68, 69), and *R. pomeroyi* growth rate in the model systems (0.95 d^−1^ for the first 4 d of the study and 0.31 d^−1^ for the 8 d average) fell within the range reported for Rhodobacteraceae in natural marine systems (70). The importance of interactions among bacteria that share ocean hot spots is underscored by the >200 *R. pomeroyi* genes with measurable effects in multispecies communities. Among the changes predicted to occur in the surface ocean environment during the coming decades, decreased nutrient supply (71–73) may intensify competition for phycosphere nitrogen and phosphorus, and altered phytoplankton community composition may affect organic carbon available to bacteria (74, 75, 76). Such changes to the arena of bacterial competition have the potential to impact carbon and nutrient biogeochemistry in the surface ocean.

## Methods

### Transposon Library Generation.

Competent *R. pomeroyi* DSS-3 cells were prepared according to Sebastián and Ammerman (23). Briefly, cells were grown at 20 °C in YTSS (4 g/L yeast, 2.5 g/L tryptone, and 20 g/L sea salts) with the addition of 40 mM sucrose to 0.4 OD_600_. The cells were washed three times with ice-cold 10% glycerol, resuspended 1:1,000 of the original culture volume in 10% glycerol, and divided into 100 µL aliquots for electroporation. A 1 µL aliquot of Ez-Tn5 <R6Kyori/KAN-2>Tn Transposome (Lucigen) was added to 100 µL of competent cells just prior to electroporation. The cells were electro-transformed in a 2-mm electrode gap cuvette using a Bio-Rad GenePulser II set to 1.8 kV, 25 mF, and 200 V, and then immediately recovered in 1 mL of YTSS at 30 °C for 2 h. The recovered cells were plated on YTSS+25 µg mL^−1^ kanamycin (KAN) agar and incubated at 30 °C for up to 72 h. Mutant colonies were counted and washed from plates using Marine Basal Medium (77), and added to glycerol at a final concentration of 20% (*SI Appendix*, Fig. S1). Aliquots were stored at −80 °C.

### Model Phycospheres.

*Ruegeria pomeroyi* DSS-3, *V. hepatarius* HF70, and *M. donghaensis* HF1 were selected for this study because they were the three most abundant members of a phycosphere enrichment culture; further, they had 16S rRNA sequences with >95% identity to bacteria known to associate with marine phytoplankton (14, 21) and survive in coculture with phytoplankton. Prior to experimentation, *V. hepatarius* and *M. donghaensis* were inoculated into YTSS medium from isolated colonies and grown overnight at 30 °C and 200 rpm. A concentrated *R. pomeroyi* mutant library cryostock was thawed on ice and inoculated at ~0.2 OD_600_ into YTSS+KAN medium and grown for ~6 h, resulting in two doubling periods. At the same time, *V. hepatarius* and *M. donghaensis* were subcultured and grown to exponential phase (0.4 to 0.6 OD_600_). Cells were pelleted at 4,000× g, washed three times, and resuspended to ~5 × 10^7^ cells mL^−1^ in 0.2 µm filter-sterilized artificial seawater (ASW). Four aliquots of washed transposon mutants were stored at −80 °C; these were used to determine the mutant library composition prior to selection.

*T. pseudonana* CCMP 1335 (National Center for Marine Algae and Microbiota) was maintained in exponential growth by diluting into fresh L1+Si ASW (78) every 4 d to a starting density of ~10^4^ cells mL^−1^ for three consecutive transfers at 20 °C with a 16 h:8 h light:dark cycle prior to experimentation. Diatom cultures were inoculated into 24 × 300 cm^3^ vented polystyrene tissue culture flasks with 500 mL L1+Si ASW, and seeded with bacteria at ~10^5^ cells mL^−1^ each to establish four treatments: *R. pomeroyi* single-bacterial culture (*Rp*), *R. pomeroyi* plus *V. hepatarius* (*Rp*+*V*), *R. pomeroyi* plus *M. donghaensis* (*Rp*+*M*), and *R. pomeroyi* plus *V. hepatarius* and *M. donghaensis* (*Rp*+*V*+*M*) (n = 4) (*SI Appendix*, Fig. S1).

### Cell Enumeration.

Bacterial abundance was determined for each species based on visually distinct colony-forming units (CFUs) on YTSS and YTSS+KAN plates on days 0, 4, and 8. Diatom abundance and combined bacterial abundance was determined by flow cytometry on a Beckman Coulter CyAn ADP (Beckman Coulter, Hialeah, Florida) on days 0, 2, 4, 6, and 8. Samples were fixed with a final concentration of 1% paraformaldehyde in the dark at room temperature for 15 min before storing at −80 °C. Samples were diluted in 0.2 µm filter-sterilized ASW, stained with 1X SYBR Green I (Thermo), and AccuCount Fluorescent particles were added as a standard. Total bacteria and phytoplankton cells were enumerated with side scatter and green and red fluorescence by a 488 nm laser applying a FL-530/30 bandpass filter and a FL4-680/30 bandpass filter. Data were analyzed in FlowJo v10.2 (Ashland, OR: Becton, Dickinson and Company; 2019) and corrected based on fluorescent particle counts as per the manufacturer’s instructions.

### Bacterial Inorganic Nitrogen Utilization Profiles and Substrate Tests.

Wild-type *R. pomeroyi, V. hepatarius,* and *M. donghaensis* were inoculated into YTSS medium and grown overnight as above. Cells were washed and transferred as monocultures supplemented with glucose (5 mM) and either ammonium (1 mM) or nitrate (1 mM), or with no nitrogen source, and grown in a 24-well plate in a Synergy H1 microplate reader (BioTek) at 30 °C with constant shaking. OD_600_ was measured every 15 min until stationary phase at approximately 60 h. Substrate tests were performed similarly, using Marine Basal Medium supplemented with ammonium and substrates glycolate, DMSP, glutamate, glutamine, asparagine, or aspartate at 12 mM carbon for 20 h.

### Validation of Purine Crossfeeding.

Washed cells from an overnight culture of WT *R. pomeroyi, V. hepatarius,* and *M. donghaensis* were transferred to L1 ASW supplemented with glucose (1 mM) and ammonium (0.5 mM), and grown to stationary phase. Cultures were centrifuged (4,000× g, 5 min), and supernatant was filtered (0.2 µm) and stored at 4 °C as cell-free spent medium. *R. pomeroyi* mutants for de novo purine synthesis (Δ*purF,* Δ*purN*) generated through transposon mutagenesis (courtesy of Dr. Christopher Reisch, University of Florida) were inoculated into YTSS and grown overnight. Cells were washed and transferred to L1 ASW supplemented with glucose (1 mM) and ammonium (0.5 mM) and six treatments were established with addition of an equal volume of cell-free spent medium of *V. hepatarius, M. donghaensis,* or wild-type *R. pomeroyi* pregrown in L1 supplemented with glucose (1 mM), an equal volume of ASW, an equal volume of ASW containing dNTPs (0.1 mM), or an equal volume of ASW containing adenosine (0.1 mM). Cells were grown in a 96-well plate in a Synergy H1 microplate reader (BioTek) at 30 °C with constant shaking, and OD_600_ was measured every 30 min for 40 h.

### DNA Extraction, Sequencing, and Bioinformatic Analysis.

After 8 d, DNA was extracted from each model phycosphere and from aliquots of the initial mutant library inoculum (n = 4) using the ZymoBIOMICS DNA Miniprep Kit (D4300) according to manufacturer’s instructions. Library preparation and sequencing was performed at the Georgia Genomics and Bioinformatics Core facility using custom PCR oligomers consisting of a region complementary to the transposon sequence with a 6 bp random heterogeneity spacer (​5′A​TGA​TAC​GGC​GAC​CAC​CGA​GAT​CTA​CAC​TCT​TTC​CCT​ACA​CGA​CGC​TCT​TCC​GAT​CTN​NNN​NNG​ACCTGCAGGCATGCAAGCTTCAG). Transposon-specific sequencing was run on an Illumina NextSeq using a single-end 75 bp high-output flow cell.

Amplified sequences were trimmed and mapped using TPP as part of TRANSIT v3.0.2 (79). In short, transposon genome junctions were identified by the sequence AGATGTGTATAAGAGACAG in the first 60 bp and trimmed to ~22 bp beginning at the site of transposon insertion. Trimmed reads were mapped to the *R. pomeroyi* DSS-3 genome (GenBank accession NC_003911) with BWA (80) in “aln” mode allowing for one mismatch, and a wig formatted file was generated indicating each site of insertion and the number of reads that mapped to it (https://doi.org/10.5281/zenodo.7489904) (81). Wig files were combined in TRANSIT using the combine wig tool. Genome sites with no insertional mutants were excluded. The transposon mutant library was composed of 83,647 unique mutants, curated to 64,365 that fell within the central 90% of a protein coding region, resulting in an average of 15 mutant strains per gene (one mutation every 50 base pairs). Reads were normalized by scaling the read count in each sample to the grand mean across all samples (Dataset S2).

### Fitness Analysis.

Relative mutant fitness (*W*) was determined following van Opijnen (26) with some alterations. Reads mapping to each unique transposon insertion were summed within a gene, averaging 423 reads per gene. Relative fitness (*W*) of mutants was calculated using the equation:

$$W=\frac{\text{ln}\left[N\left(t2\right)*\frac{d}{N\left(t1\right)}+1\right]}{\text{ln}\left[\left(1-N\left(t2\right)\right)*\frac{d}{\left(1-N\left(t1\right)\right)}+1\right]},$$

where *N*(*t*1) and *N*(*t*2) are the frequencies of a mutant in the pool at the start and end of the experiment, respectively, and d (expansion factor) represents the fold change of the total mutant pool during the 8-d growth period. Expansion factors and generation times were calculated based on flow cytometry data (cells mL^−1^), using CFU data to determine the proportion of *R. pomeroyi* in the bacterial community. Mutants with <10 average reads between the initial and final time points were removed from the analysis.

The mean relative fitness for 3,748 out of 4,293 coding regions (Dataset S3) was determined from four replicate measurements of fitness. Fitness differences were calculated as:

$$\log_{2}\left(\frac{W_{multi-bacteria}+\Psi }{W_{Rp}+\Psi }\right),$$

where Ψ is a small number (0.01) to prevent a zero or noninteger. Statistical significance of fitness differences was determined by randomization tests (82) with 10,000 permutations using a custom script (83), and *P*-values were adjusted for multiple comparisons using the Benjamini–Hochberg procedure within the stats (v3.6.2) package in R. Mutant fitness analysis assumed that phytoplankton growth and release of photosynthate was independent of bacterial strains present, consistent with similar diatom growth (Fig. 1*B*) and comparable photosynthetic physiology (*SI Appendix*, Fig. S2) in all treatments.

All plots and statistical analyses were performed in R v4.0.1 (84) using the packages tidyverse (85) and data.table (86). Figures were generated in R and Adobe Illustrator 2020.

## Supplementary Material

Appendix 01 (PDF)

Dataset S01 (XLSX)

Dataset S02 (XLSX)

Dataset S03 (XLSX)

## Data Availability

Code for analyzing data is available at github.com/jschreie/TnSeq_Phycosphere_Interactions (87). FASTQ Transposon sequencing files, all code and data used to analyze the data, wig formatted file output from TRANSIT with all transposon insertions data have been deposited in NCBI BioProject with accession no. PRJNA910220 (88); GitHub (87); Zenodo (81, 89). All other data are included in the manuscript and/or *SI Appendix*.

## References

[1] M. J. Behrenfeld, E. Boss, D. A. Siegel, D. M. Shea, Carbon-based ocean productivity and phytoplankton physiology from space. Glob. Biogeochem. Cycles **19** (2005).

[2] M. A. Moran , The ocean’s labile DOC supply chain. Limnol. Oceanogr. **67**, 1007–1021 (2022).

[3] D. A. Hansell, Recalcitrant dissolved organic carbon fractions. Ann. Rev. Mar. Sci. **5**, 421–445 (2013). 10.1146/annurev-marine-120710-100757 22881353

[4] F. Azam, F. Malfatti, Microbial structuring of marine ecosystems. Nat. Rev. Microbiol. **5**, 782–791 (2007). 17853906 10.1038/nrmicro1747

[5] M. A. Moran , Microbial metabolites in the marine carbon cycle. Nat. Microbiol. **7**, 508–523 (2022). 35365785 10.1038/s41564-022-01090-3

[6] W. Bell, R. Mitchell, Chemotactic and growth responses of marine bacteria to algal extracellular products. Biol. Bull. **143**, 265–277 (1972).

[7] J. R. Seymour, S. A. Amin, J. B. Raina, R. Stocker, Zooming in on the phycosphere: the ecological interface for phytoplankton-bacteria relationships. Nat. Microbiol. **2**, 17065 (2017). 28555622 10.1038/nmicrobiol.2017.65

[8] S. A. Amin, M. S. Parker, E. V. Armbrust, Interactions between diatoms and bacteria. Microbiol. Mol. Biol. Rev. **76**, 667–684 (2012). 22933565 10.1128/MMBR.00007-12 PMC3429620

[9] M. Landa , Sulfur metabolites that facilitate oceanic phytoplankton-bacteria carbon flux. ISME J. **13**, 2536–2550 (2019). 31227817 10.1038/s41396-019-0455-3 PMC6776065

[10] A. A. Shibl , Diatom modulation of select bacteria through use of two unique secondary metabolites. Proc. Natl. Acad. Sci. U.S.A. **117**, 27445–27455 (2020). 33067398 10.1073/pnas.2012088117 PMC7959551

[11] H. P. Grossart, F. Levold, M. Allgaier, M. Simon, T. Brinkhoff, Marine diatom species harbour distinct bacterial communities. Environ. Microbiol. **7**, 860–873 (2005). 15892705 10.1111/j.1462-2920.2005.00759.x

[12] G. Behringer , Bacterial communities of diatoms display strong conservation across strains and time. Front. Microbiol. **9**, 659 (2018). 29681892 10.3389/fmicb.2018.00659 PMC5897529

[13] M. M. Barreto Filho, M. Walker, M. P. Ashworth, J. J. Morris, Structure and long-term stability of the microbiome in diverse diatom cultures. Microbiol. Spectr. **9**, e00269–00221 (2021). 34190604 10.1128/spectrum.00269-21 PMC8552671

[14] H. Fu, M. Uchimiya, J. Gore, M. A. Moran, Ecological drivers of bacterial community assembly in synthetic phycospheres. Proc. Natl. Acad. Sci. U.S.A. **117**, 3656–3662 (2020). 32015111 10.1073/pnas.1917265117 PMC7035482

[15] H. Teeling , Substrate-controlled succession of marine bacterioplankton populations induced by a phytoplankton bloom. Science **336**, 608–611 (2012). 22556258 10.1126/science.1218344

[16] K. Krüger , In marine *Bacteroidetes* the bulk of glycan degradation during algae blooms is mediated by few clades using a restricted set of genes. ISME J. **13**, 2800–2816 (2019). 31316134 10.1038/s41396-019-0476-y PMC6794258

[17] D. M. Needham, J. A. Fuhrman, Pronounced daily succession of phytoplankton, archaea and bacteria following a spring bloom. Nat. Microbiol. **1**, 1–7 (2016). 10.1038/nmicrobiol.2016.5 27572439

[18] A. M. Martin-Platero , High resolution time series reveals cohesive but short-lived communities in coastal plankton. Nat. Commun. **9**, 1–11 (2018). 29348571 10.1038/s41467-017-02571-4 PMC5773528

[19] A. Buchan, G. R. LeCleir, C. A. Gulvik, J. M. González, Master recyclers: Features and functions of bacteria associated with phytoplankton blooms. Nat. Rev. Microbiol. **12**, 686–698 (2014). 25134618 10.1038/nrmicro3326

[20] R. Stocker, Marine microbes see a sea of gradients. Science **338**, 628–633 (2012). 23118182 10.1126/science.1208929

[21] H. Fu, C. B. Smith, S. Sharma, M. A. Moran, Genome sequences and metagenome-assembled genome sequences of microbial communities enriched on phytoplankton exometabolites. Microbiol. Resour. Announce. **9**, e00724–24 (2020). 10.1128/MRA.00724-20 PMC7378039 32703840

[22] J. M. González, R. P. Kiene, M. A. Moran, Transformation of sulfur compounds by an abundant lineage of marine bacteria in the α-subclass of the class proteobacteria. Appl. Environ. Microbiol. **65**, 3810–3819 (1999). 10473380 10.1128/aem.65.9.3810-3819.1999 PMC99705

[23] M. Sebastián, J. Ammerman, Role of the phosphatase PhoX in the phosphorus metabolism of the marine bacterium *Ruegeria pomeroyi* DSS-3. Environ. Microbiol. Rep. **3**, 535–542 (2011). 23761332 10.1111/j.1758-2229.2011.00253.x

[24] G. C. Sharpe, S. M. Gifford, A. N. Septer, A model roseobacter, *Ruegeria pomeroyi* DSS-3, employs a diffusible killing mechanism to eliminate competitors. mSystems **5**, e00443–00420 (2020). 32788406 10.1128/mSystems.00443-20 PMC7426152

[25] M. C. Chao, S. Abel, B. M. Davis, M. K. Waldor, The design and analysis of transposon insertion sequencing experiments. Nat. Rev. Microbiol. **14**, 119–128 (2016). 26775926 10.1038/nrmicro.2015.7 PMC5099075

[26] T. van Opijnen, K. L. Bodi, A. Camilli, Tn-seq: High-throughput parallel sequencing for fitness and genetic interaction studies in microorganisms. Nat Methods **6**, 767–772 (2009). 19767758 10.1038/nmeth.1377 PMC2957483

[27] T. R. Ioerger, Analysis of gene essentiality from TnSeq data using transit. Methods Mol. Biol. **2377**, 391–421 (2022). 34709629 10.1007/978-1-0716-1720-5_22 PMC8941984

[28] M. A. Moran , Genome sequence of *Silicibacter pomeroyi* reveals adaptations to the marine environment. Nature **432**, 910–913 (2004). 15602564 10.1038/nature03170

[29] L. Reitzer, Nitrogen assimilation and global regulation in *Escherichia coli*. Annu. Rev. Microbiol. **57**, 155–176 (2003). 12730324 10.1146/annurev.micro.57.030502.090820

[30] P. M. Glibert , Pluses and minuses of ammonium and nitrate uptake and assimilation by phytoplankton and implications for productivity and community composition, with emphasis on nitrogen-enriched conditions. Limnol. Oceanogr. **61**, 165–197 (2016).

[31] A. E. Allen , Diversity and detection of nitrate assimilation genes in marine bacteria. Appl. Environ. Microbiol. **67**, 5343–5348 (2001). 11679368 10.1128/AEM.67.11.5343-5348.2001 PMC93313

[32] D. L. Kirchman, P. A. Wheeler, Uptake of ammonium and nitrate by heterotrophic bacteria and phytoplankton in the sub-Arctic Pacific. Deep Sea Res Part I Oceanogr. Res. Pap. **45**, 347–365 (1998).

[33] S. Lockwood, C. Greening, F. Baltar, S. E. Morales, Global and seasonal variation of marine phosphonate metabolism. ISME J. **16**, 2198–2212 (2022). 35739297 10.1038/s41396-022-01266-z PMC9381506

[34] K. Ishige, H. Zhang, A. Kornberg, Polyphosphate kinase (PPK2), a potent, polyphosphate-driven generator of GTP. Proc. Natl. Acad. Sci. U.S.A. **99**, 16684–16688 (2002). 12482933 10.1073/pnas.262655299 PMC139204

[35] H. Luo, M. A. Moran, How do divergent ecological strategies emerge among marine bacterioplankton lineages? Trends Microbiol. **23**, 577–584 (2015). 26051014 10.1016/j.tim.2015.05.004

[36] Ö. Özkaya, K. B. Xavier, F. Dionisio, R. Balbontín, Maintenance of microbial cooperation mediated by public goods in single-and multiple-trait scenarios. J. Bacteriol. **199**, e00297–00217 (2017). 28847922 10.1128/JB.00297-17 PMC5648865

[37] S. A. Sañudo-Wilhelmy , Multiple B-vitamin depletion in large areas of the coastal ocean. Proc. Natl. Acad. Sci. U.S.A. **109**, 14041–14045 (2012). 22826241 10.1073/pnas.1208755109 PMC3435217

[38] L. Gómez-Consarnau , Mosaic patterns of B-vitamin synthesis and utilization in a natural marine microbial community. Environ. Microbiol. **20**, 2809–2823 (2018). 29659156 10.1111/1462-2920.14133

[39] S. Pontrelli , Metabolic cross-feeding structures the assembly of polysaccharide degrading communities. Sci. Adv. **8**, eabk3076 (2022). 35196097 10.1126/sciadv.abk3076 PMC8865766

[40] G. D’Souza , Ecology and evolution of metabolic cross-feeding interactions in bacteria. Nat. Prod. Rep. **35**, 455–488 (2018). 29799048 10.1039/c8np00009c

[41] K. Amarnath , Dynamic metabolic exchanges between complementary bacterial types provide collaborative stress resistance. bioXRiv [Preprint] (2022). Accessed 1 December 2022.

[42] B. LaSarre, A. M. Deutschbauer, C. E. Love, J. B. McKinlay, Covert cross-feeding revealed by genome-wide analysis of fitness determinants in a synthetic bacterial mutualism. Appl. Environ. Microbiol. **86**, e00543-20 (2020). 32332139 10.1128/AEM.00543-20 PMC7301861

[43] M. D. Linney, C. R. Schvarcz, G. F. Steward, E. F. DeLong, D. M. Karl, A method for characterizing dissolved DNA and its application to the North Pacific Subtropical Gyre. Limnol. Oceanogr. Methods **19**, 210–221 (2021).

[44] J. Kehe , Positive interactions are common among culturable bacteria. Sci. Adv. **7**, eabi7159 (2020). 10.1126/sciadv.abi7159 PMC8570599 34739314

[45] A. Zelezniak , Metabolic dependencies drive species co-occurrence in diverse microbial communities. Proc. Natl. Acad. Sci. U.S.A. **112**, 6449–6454 (2015). 25941371 10.1073/pnas.1421834112 PMC4443341

[46] G. D’Souza , Less is more: Selective advantages can explain the prevalent loss of biosynthetic genes in bacteria. Evolution **68**, 2559–2570 (2014). 24910088 10.1111/evo.12468

[47] J. E. Goldford , Emergent simplicity in microbial community assembly. Science **361**, 469–474 (2018). 30072533 10.1126/science.aat1168 PMC6405290

[48] H. Ploug, M. Kühl, B. Buchholz-Cleven, B. B. Jørgensen, Anoxic aggregates-an ephemeral phenomenon in the pelagic environment? Aquat. Microb. Ecol. **13**, 285–294 (1997).

[49] I. Klawonn, S. Bonaglia, V. Brüchert, H. Ploug, Aerobic and anaerobic nitrogen transformation processes in N_2_-fixing cyanobacterial aggregates. ISME J **9**, 1456–1466 (2015). 25575306 10.1038/ismej.2014.232 PMC4438332

[50] A. L. Alldredge, Y. Cohen, Can microscale chemical patches persist in the sea? Microelectrode study of marine snow, fecal pellets. Science **235**, 689–691 (1987). 17833630 10.1126/science.235.4789.689

[51] I. Wagner-Döbler, H. Biebl, Environmental biology of the marine *Roseobacter* lineage. Annu. Rev. Microbiol. **60**, 255–280 (2006). 16719716 10.1146/annurev.micro.60.080805.142115

[52] R. J. Newton , Genome characteristics of a generalist marine bacterial lineage. ISME J. **4**, 784–798 (2010). 20072162 10.1038/ismej.2009.150

[53] E. R. Zinser, The microbial contribution to reactive oxygen species dynamics in marine ecosystems. Environ. Microbiol. Rep. **10**, 412–427 (2018). 29411545 10.1111/1758-2229.12626

[54] J. M. Diaz, S. Plummer, Production of extracellular reactive oxygen species by phytoplankton: Past and future directions. J. Plankton Res. **40**, 655–666 (2018). 30487658 10.1093/plankt/fby039 PMC6247811

[55] C. M. Hansel, J. M. Diaz, S. Plummer, Tight regulation of extracellular superoxide points to its vital role in the physiology of the globally relevant Roseobacter clade. mBio **10**, e02668–02618 (2019). 10.1128/mBio.02668-18 PMC6414704 30862752

[56] J. M. Diaz , Widespread production of extracellular superoxide by heterotrophic bacteria. Science **340**, 1223–1226 (2013). 23641059 10.1126/science.1237331

[57] T. Oda , Generation of reactive oxygen species by raphidophycean phytoplankton. Bioscience **61**, 1658–1662 (1997). 10.1271/bbb.61.1658 9362113

[58] M. Scott, S. Klumpp, E. M. Mateescu, T. Hwa, Emergence of robust growth laws from optimal regulation of ribosome synthesis. Mol. Syst. Biol. **10**, 747 (2014). 25149558 10.15252/msb.20145379 PMC4299513

[59] S. Klumpp, M. Scott, S. Pedersen, T. Hwa, Molecular crowding limits translation and cell growth. Proc. Natl. Acad. Sci. U.S.A. **110**, 16754–16759 (2013). 24082144 10.1073/pnas.1310377110 PMC3801028

[60] E. Bosdriesz, D. Molenaar, B. Teusink, F. J. Bruggeman, How fast-growing bacteria robustly tune their ribosome concentration to approximate growth-rate maximization. FEBS J. **282**, 2029–2044 (2015). 25754869 10.1111/febs.13258 PMC4672707

[61] U. Sauer, F. Canonaco, S. Heri, A. Perrenoud, E. Fischer, The soluble and membrane-bound transhydrogenases UdhA and PntAB have divergent functions in NADPH metabolism of *Escherichia coli*. J. Biol. Chem. **279**, 6613–6619 (2004). 14660605 10.1074/jbc.M311657200

[62] G. M. Cook, K. Hards, C. Vilchèze, T. Hartman, M. Berney, Energetics of respiration and oxidative phosphorylation in mycobacteria. Microbiol. Spectr. **2**, 06 (2014). 10.1128/microbiolspec.MGM2-0015-2013 PMC4205543 25346874

[63] M. Cunliffe, Physiological and metabolic effects of carbon monoxide oxidation in the model marine bacterioplankton *Ruegeria pomeroyi* DSS-3. Appl. Environ. Microbiol. **79**, 738–740 (2013). 23144131 10.1128/AEM.02466-12 PMC3553778

[64] J. A. Christie-Oleza, B. Fernandez, B. Nogales, R. Bosch, J. Armengaud, Proteomic insights into the lifestyle of an environmentally relevant marine bacterium. ISME J. **6**, 124–135 (2012). 21776030 10.1038/ismej.2011.86 PMC3246242

[65] P. R. Cordero , Atmospheric carbon monoxide oxidation is a widespread mechanism supporting microbial survival. ISME J. **13**, 2868–2881 (2019). 31358912 10.1038/s41396-019-0479-8 PMC6794299

[66] E. Kazamia, K. E. Helliwell, S. Purton, A. G. Smith, How mutualisms arise in phytoplankton communities: building eco-evolutionary principles for aquatic microbes. Ecol. Lett. **19**, 810–822 (2016). 27282316 10.1111/ele.12615 PMC5103174

[67] J. D. Palmer, K. R. Foster, Bacterial species rarely work together. Science **376**, 581–582 (2022). 35511986 10.1126/science.abn5093

[68] L. Riemann, G. F. Steward, F. Azam, Dynamics of bacterial community composition and activity during a mesocosm diatom bloom. Appl. Environ. Microbiol. **66**, 578–587 (2000). 10653721 10.1128/aem.66.2.578-587.2000 PMC91866

[69] B. Norrman, U. L. Zwelfel, C. S. Hopkinson Jr., F. Brian, Production and utilization of dissolved organic carbon during an experimental diatom bloom. Limnol. Oceanogr. **40**, 898–907 (1995).

[70] S. M. Gifford, S. Sharma, M. Booth, M. A. Moran, Expression patterns reveal niche diversification in a marine microbial assemblage. ISME J. **7**, 281–298 (2013). 22931830 10.1038/ismej.2012.96 PMC3554409

[71] P. W. Boyd , Biological responses to environmental heterogeneity under future ocean conditions. Glob. Chang Biol. **22**, 2633–2650 (2016). 27111095 10.1111/gcb.13287

[72] J. Beardall, S. Stojkovic, S. Larsen, Living in a high CO_2_ world: Impacts of global climate change on marine phytoplankton. Plant Ecol. Divers **2**, 191–205 (2009).

[73] K. Gao, E. W. Helbling, D.-P. Häder, D. A. Hutchins, Responses of marine primary producers to interactions between ocean acidification, solar radiation, and warming. Mar. Ecol. Prog. Ser. **470**, 167–189 (2012).

[74] D. B. Van de Waal, E. Litchman, Multiple global change stressor effects on phytoplankton nutrient acquisition in a future ocean. Philos. Trans. R Soc. Lond. B Biol. Sci. **375**, 20190706 (2020). 32200734 10.1098/rstb.2019.0706 PMC7133525

[75] J. A. Hellebust, Excretion of some organic compounds by marine phytoplankton. Limnol. Oceanogr. **10**, 192–206 (1965).

[76] D. C. O. Thornton, Dissolved organic matter (DOM) release by phytoplankton in the contemporary and future ocean. Eur. J. Phycol. **49**, 20–46 (2014).

[77] P. Baumann, "The marine gram negative eubacteria: Genera *Photobacterium, Benekea, Alteromonas*, *Pseudomonsa* and *Alcaligenes*” in The prokaryotes, M. P. Stolp, H. Trüper, H. G. Balows, A. Schlegel, Eds. (Springer-Verlag, 1981), pp. 1302–1331.

[78] J. A. Berges, D. J. Franklin, P. J. Harrison, Evolution of an artificial seawater medium: improvements in enriched seawater, artificial water over the last two decades. J. Phycol. **37**, 1138–1145 (2001).

[79] M. A. DeJesus, C. Ambadipudi, R. Baker, C. Sassetti, T. R. Ioerger, TRANSIT—a software tool for himar1 TnSeq analysis. PloS Comput. Biol. **11**, e1004401 (2015). 26447887 10.1371/journal.pcbi.1004401 PMC4598096

[80] H. Li, R. Durbin, Fast and accurate short read alignment with Burrows-Wheeler transform. Bioinformatics **25**, 1754–1760 (2009). 19451168 10.1093/bioinformatics/btp324 PMC2705234

[81] J. E. Schreier , Wig formatted file. Zenodo. 10.5281/zenodo.7489904. Deposited 28 December 2022.

[82] P. H. Crowley, Resampling methods for computation-intensive data analysis in ecology and evolution. Annu. Rev. Ecol. Evol. Syst. **23**, 405–447 (1992).

[83] S. Holland, Data analysis in the geosciences. http://strata.uga.edu/8370/lecturenotes. Accessed 6 June 2022.

[84] R Core Team, R: A language and environment for statistical computing (Version 4.1.1., R Foundation for Statistical Computing, Vienna, Austria, 2019).

[85] H. Wickham , Welcome to the Tidyverse Version 2.0.0. https://tidyverse.tidyverse.org/articles/paper.html. Accessed 1 December 2022.

[86] M. Dowle , data.table: Extension of ‘data.frame’ Version 1.14.8. https://cran.r-project.org/web/packages/data.table/index.html. Accessed 1 December 2022.

[87] J. E. Schreier, jschreie/TnSeq_Phycosphere_Interactions. Github. https://github.com/jschreie/TnSeq_Phycosphere_Interactions. Deposited 1 December 2022.

[88] J. E. Schreier , Model Diatom Phycosphere TnSeq. NCBI BioProject. https://www.ncbi.nlm.nih.gov/bioproject/910220. Deposited 8 December 2022

[89] J. E. Schreier, TnSeq_Phycosphere_Interactions v1.1.1. Zenodo. 10.5281/zenodo.7489891. Deposited 28 December 2022.

