# Supplementary material for: A mutant fitness assay identifies bacterial interactions in a model ocean hot spot
Source: Proc Natl Acad Sci U S A. 2023 Mar 15;120(12):e2217200120. doi: 10.1073/pnas.2217200120 (PMC10041152; doi:10.1073/pnas.2217200120)
Supplement: Supplementary file 1 — Appendix 01 (PDF) [file pnas.2217200120.sapp.pdf]

PNAS

Supporting Information for
A Mutant Fitness Assay Identifies Bacterial Interactions in a Model Ocean Hot-Spot

Jeremy E. Schreier, Christa B. Smith, Thomas R. Ioerger, Mary Ann Moran
Jeremy E. Schreier
Email: Jeremy.e.schreier@gmail.com

This PDF file includes:

Figures S1 to S5
Tables S1 to S3
Dataset S1 to S3

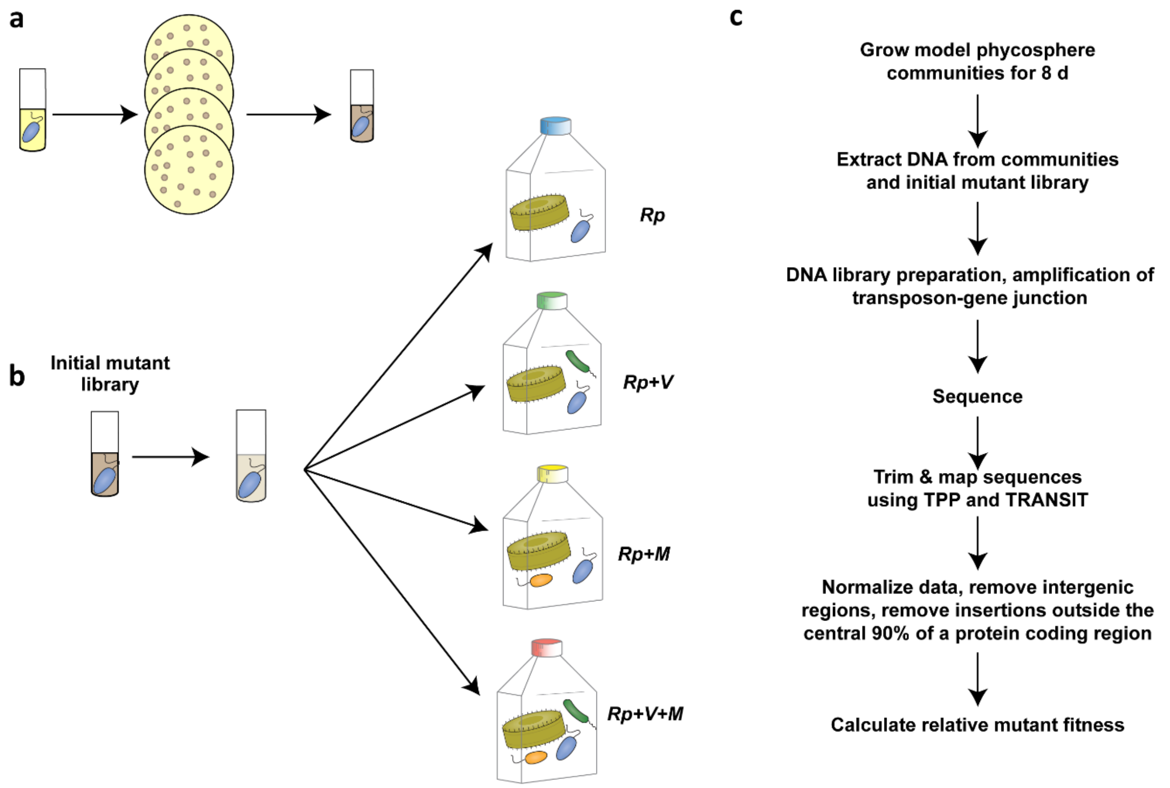

Supplemental Figure S1. Graphical explanation of transposon mutant library generation, model phycosphere assay, and data processing. a. Generation of a Tn5 transposon mutant library in *Ruegeria pomeroyi* DSS-3. Electrocompetent cells were transformed with Tn5 transposome, recovered in rich medium, and plated and incubated on selective (kanamycin) medium. Colonies were washed from the plates and added to glycerol to generate a saturated transposon mutant library. b. Model phycosphere set-up. The saturated transposon mutant library of *R. pomeroyi* was inoculated into selective liquid medium, grown, washed, and resuspended to ~5x10^{7} cells ml^{-1} to generate the initial mutant library, which was inoculated into diatom cultures to establish four treatments: *R. pomeroyi* single-bacterial culture (*Rp*), *R. pomeroyi* plus *Vibrio hepatarius* HF70 (*Rp*+*V*), *R.pomeroyi* plus *Marivivens donghaensis* HF1 (*Rp*+*M*), and *R. pomeroyi* plus *V. hepatarius* plus *M. donghaensis* (*Rp*+*V*+*M*) (n=4). c. Data generation and processing. Model phycosphere communities were grown for 8 d. Cultures were pelleted, and DNA was extracted from each community and the initial mutant library. DNA libraries were prepared for transposon sequencing, and subsequently processed to calculate relative mutant fitness.

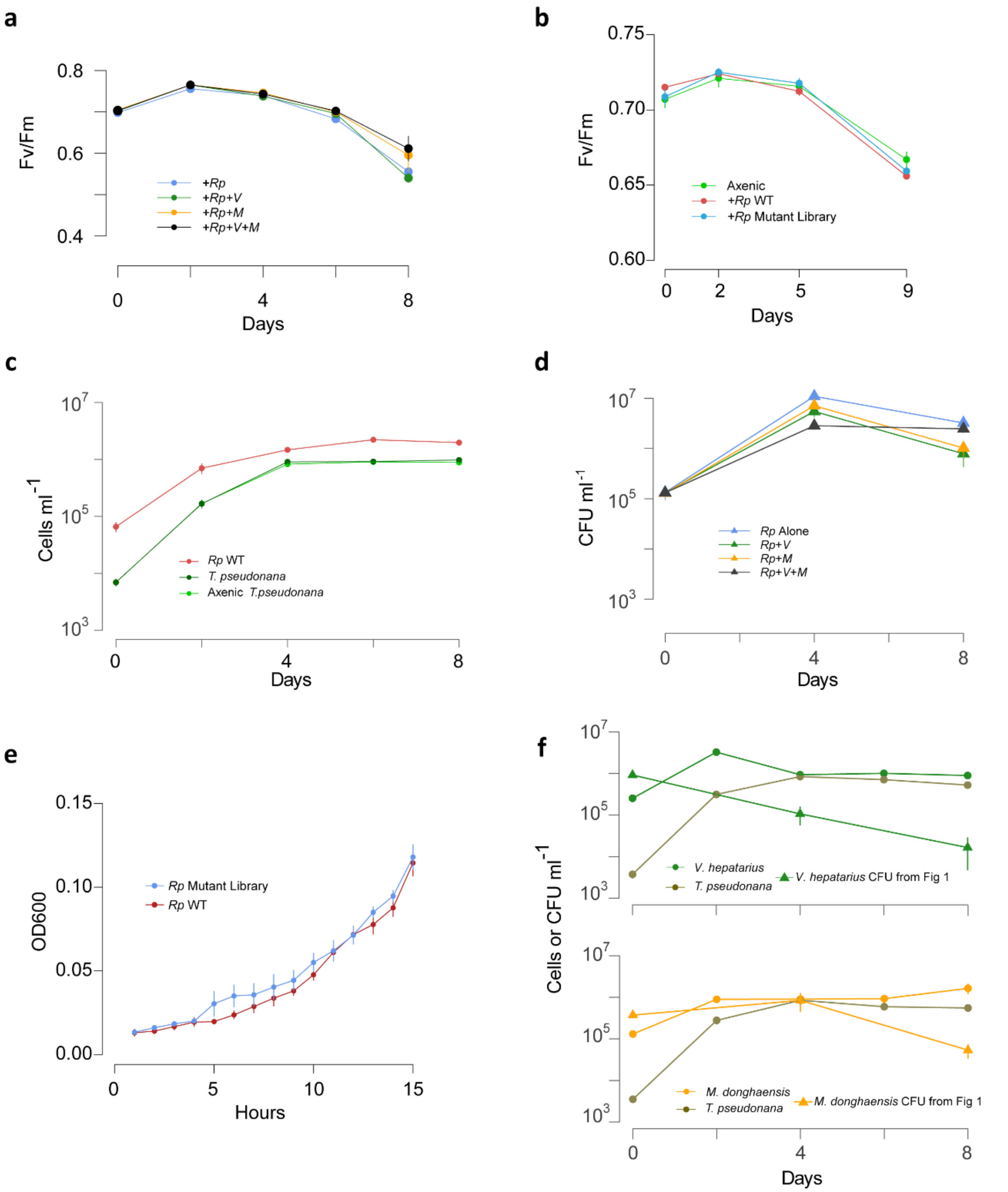

Supplemental Figure S2. Microbial growth and physiology. Diatom photosynthetic efficiency (Fv/Fm) measured by a Satlantic FIRe Fluorometer. (a) Mean Fv/Fm measured for model phycosphere communities and (b) communities containing axenic diatoms or co-cultures with wild-type *R. pomeroyi* or the mutant library. Mean Fv/Fm at the final time points are statistically indistinguishable (a; F=1.7, df=3, p=0.22. b; F=3.1, df=2, p=0.12). (c) Growth of *T. pseudonana* in coculture with wild-type *R. pomeroyi* and axenic (n=3). (d) Growth of the *R. pomeroyi* mutant library in each of the four model phycosphere communities from Figure 1b. (e) Similarity of mutant library and wild-type *R.pomeroyi* growth on ectoine (1.25 mM carbon) (n=3). (f) Cocultures of *T. pseudonana* with *V. hepatarius* (top) or *M. donghaensis* (bottom) demonstrating growth of these bacteria in the absence of *R. pomeroyi* (n=3). Mean CFUs of these bacteria from Fig 1 in the presence of *R. pomeroyi* are displayed for comparison. Some standard error bars fall within the symbol. Rp=*Ruegeria pomeroyi*, V=*Vibrio hepatarius* HF70, M=*Marivivens donghaensis* HF1.

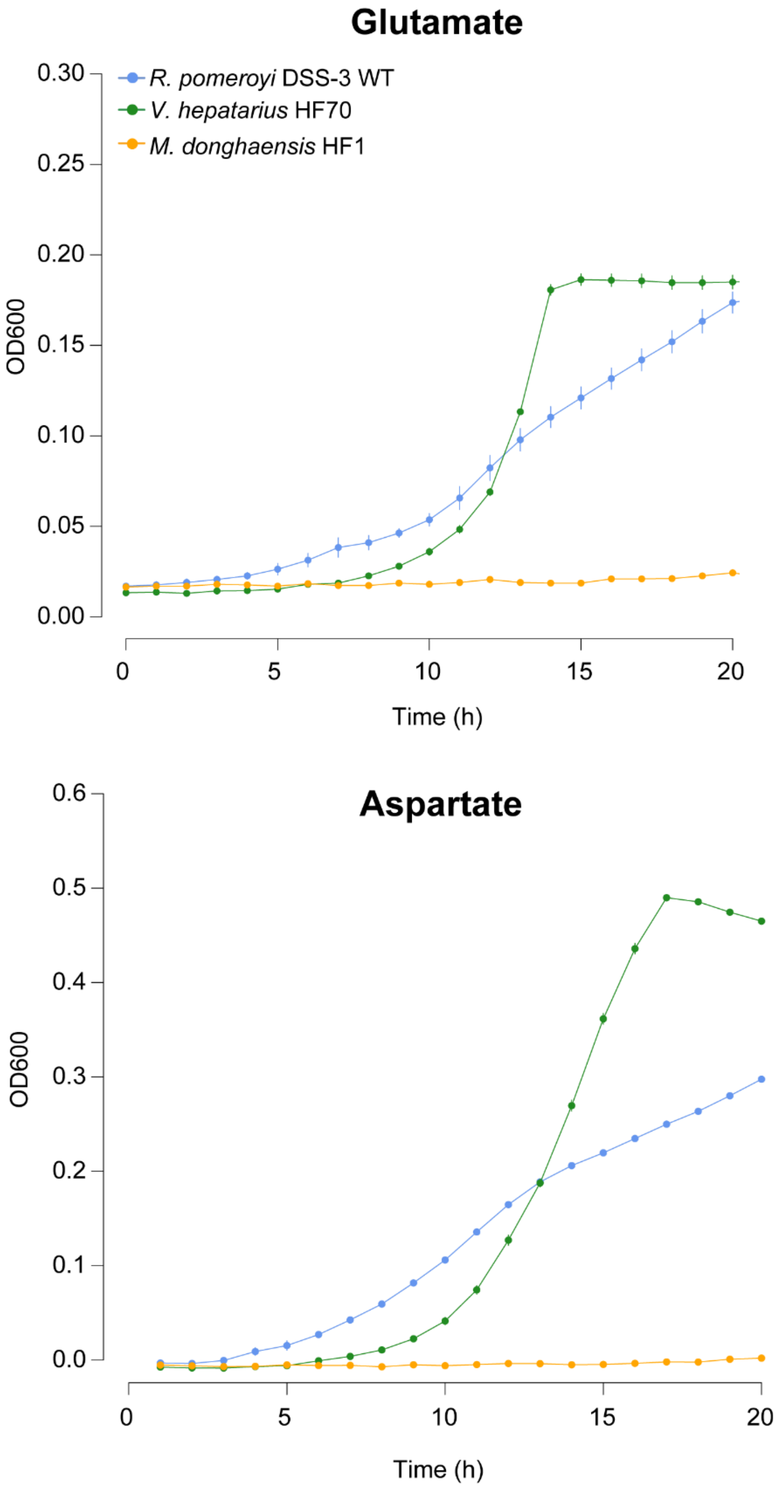

Supplemental Figure S3. Growth of monocultures in minimal medium with either glutamate or aspartate (12 mM carbon) as the sole source of carbon (n=3-6).

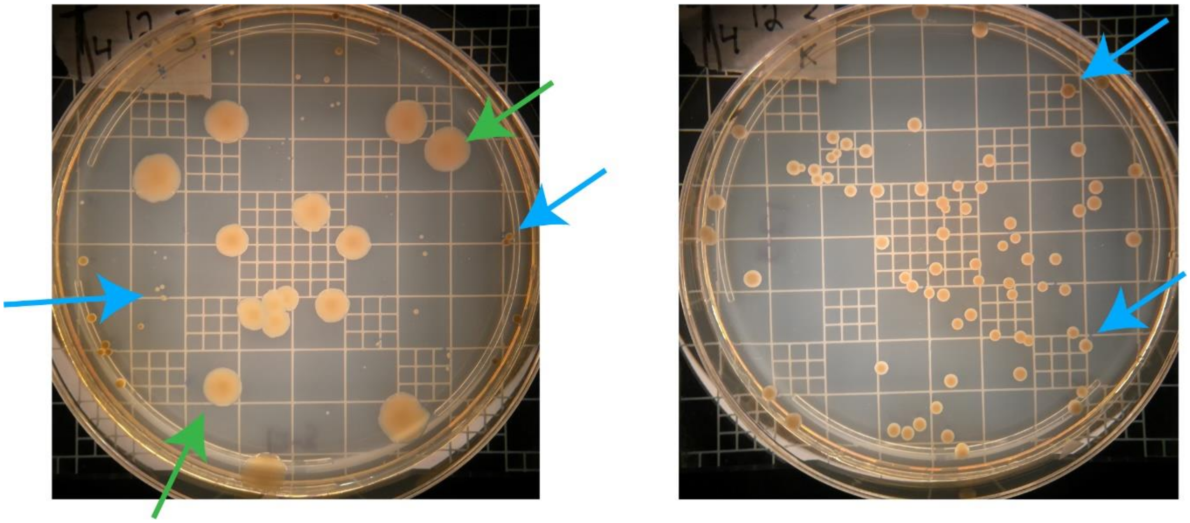

Supplemental Figure S4. Examples of non-selective (left) and selective (right, kanamycin) agar plates with CFUs of *R. pomeroyi* (blue arrows) and *V. hepatarius* (green arrows). *V. hepatarius* inhibits the growth of *R. pomeroyi* on solid medium, as seen by the clear zone of inhibition around *V. hepatarius* colonies. *M. donghaensis* does not inhibit, and is not inhibited by, other strains on solid medium.

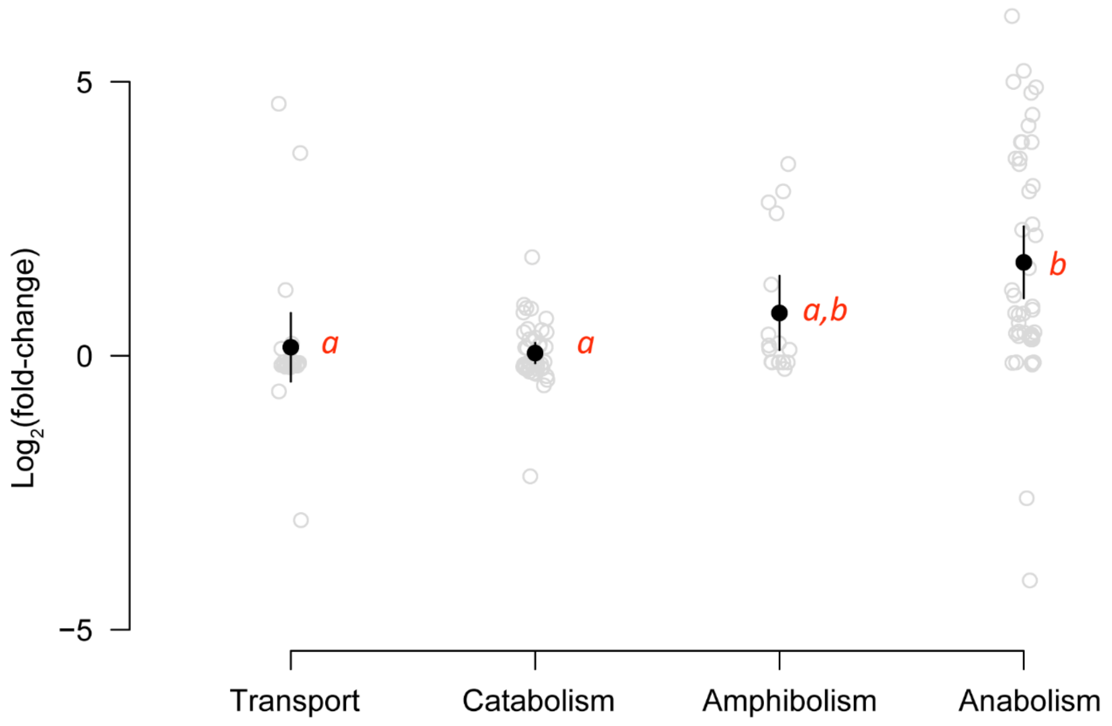

Supplemental Figure S5. Fitness differences for *R. pomeroyi* mutants with COG classifications that function in transport, catabolism, amphibolism (both catabolism and anabolism), or anabolism. Gray points are significant fitness changes in multi-bacterial communities; black points indicate the mean of significant fitness differences with 95% confidence intervals. Significant differences between categories are indicated by red letters *a* and *b.*

Table S1. IMG search results for metabolic potential of *M. donghaensis*, *V. hepatarius*, and *R. pomeroyi.*

Metabolic Process	Gene ID	Locus Tag	Gene Product Name	Genome Name
Assimilatory nitrate reduction	2889571834	Ga0440170_01_84031_86631	assimilatory nitrate reductase catalytic subunit	Marivivens donghaensis HF1
Nitrate/nitrite transport	2889571837	Ga0440170_01_89404_91083	nitrate/nitrite transport system ATP-binding protein	Marivivens donghaensis HF1
Nitrate/nitrite transport	2889571838	Ga0440170_01_91089_92171	nitrate/nitrite transport system permease protein	Marivivens donghaensis HF1
Nitrate/nitrite transport	2889571839	Ga0440170_01_92231_93598	nitrate/nitrite transport system substrate-binding protein	Marivivens donghaensis HF1
Ammonium assimilation	2889571852	Ga0440170_01_105083_106435	glutamine synthetase	Marivivens donghaensis HF1
Ammonium assimilation	2889573122	Ga0440170_17_148076_149503	glutamate synthase (NADPH/NADH) small chain	Marivivens donghaensis HF1
Ammonium assimilation	2889573124	Ga0440170_17_150233_154774	glutamate synthase (NADPH/NADH) large chain	Marivivens donghaensis HF1
Ammonium assimilation	2889572678	Ga0440170_11_280636_282021	glutamate dehydrogenase (NAD(P)+)	Marivivens donghaensis HF1
Dissimilatory nitrate reduction	2916743923	Ga0481171_06_79626_79805	nitrate reductase NapE	Vibrio hepatarius HF70
Ammonium assimilation	2916743332	Ga0481171_01_155577_156986	glutamine synthetase	Vibrio hepatarius HF70
Ammonium assimilation	2916743504	Ga0481171_03_38999_43462	glutamate synthase (NADPH/NADH) large chain	Vibrio hepatarius HF70
Ammonium assimilation	2916743505	Ga0481171_03_43483_44895	glutamate synthase (NADPH/NADH) small chain	Vibrio hepatarius HF70
Ammonium assimilation	2916745099	Ga0481171_17_51425_56266	glutamate dehydrogenase	Vibrio hepatarius HF70
Ammonium assimilation	637289773	SPO2295	L-glutamine synthetase (EC 6.3.1.2)	Ruegeria pomeroyi DSS-3
Ammonium assimilation	637289224	SPO1743	glutamate dehydrogenase	Ruegeria pomeroyi DSS-3
Ammonium assimilation	637291236	SPO3768	glutamate synthase (NADPH) large subunit (EC 1.4.1.13)	Ruegeria pomeroyi DSS-3
Ammonium assimilation	637291238	SPO3770	glutamate synthase (NADPH) small subunit (EC 1.4.1.13)	Ruegeria pomeroyi DSS-3
C-P lyase: substrate specific	2889572014	Ga0440170_03_48139_48480	protein PhnA	Marivivens donghaensis HF1
C-P lyase: substrate specific	2889572397	Ga0440170_11_12863_13279	PhnB protein	Marivivens donghaensis HF1
C-P lyase: substrate specific	2916743366	Ga0481171_02_22514_23629	2-aminoethylphosphonate-pyruvate transaminase	Vibrio hepatarius HF70
C-P lyase: broad specificity	637287960	SPO0468	alkylphosphonate utilization protein PhnG	Ruegeria pomeroyi DSS-3
C-P lyase: broad specificity	637287961	SPO0469	alkylphosphonate utilization protein PhnH	Ruegeria pomeroyi DSS-3
C-P lyase: broad specificity	637287962	SPO0470	alkylphosphonate utilization protein PhnI	Ruegeria pomeroyi DSS-3
C-P lyase: broad specificity	637287963	SPO0471	alkylphosphonate utilization protein PhnJ	Ruegeria pomeroyi DSS-3

C-P lyase: broad specificity	637287964	SPO0472	alkylphosphonate utilization protein PhnK	Ruegeria pomeroyi DSS-3
C-P lyase: broad specificity	637287965	SPO0473	alkylphosphonate utilization protein PhnL	Ruegeria pomeroyi DSS-3
C-P lyase: broad specificity	637287966	SPO0474	alkylphosphonate utilization protein PhnN	Ruegeria pomeroyi DSS-3
C-P lyase: broad specificity	637287968	SPO0476	alkylphosphonate utilization protein PhnM	Ruegeria pomeroyi DSS-3
C-P lyase: broad specificity	637288378	SPO0891	alkylphosphonate utilization protein PhnM, putative	Ruegeria pomeroyi DSS-3

Table S2. Mean generations of *R. pomeroyi* realized over the first 4 days of culture

**Community**	**Generations over 4 days**	**standard deviation**
Rp	6.4	0.13
Rp+V	5.4	0.12
Rp+M	5.8	0.06
Rp+V+M	4.4	0.09

Table S3. Statistics associated with transposon insertion reads that were trimmed and mapped to the *R. pomeroyi* genome from TRANSIT TPP output

Condition	Rep	Total Reads	Trimmed Reads	Mapped Reads	Percent Mapped	Insertion Sites
Initial Library	1	11273142	4360270	3857733	0.8847	49548
Initial Library	2	8118575	3620276	3161913	0.8734	52877
Initial Library	3	6893616	3030343	2626152	0.8666	55549
Initial Library	4	8915564	4357750	3780643	0.8676	59618
Rp	1	10970318	812423	683144	0.8409	46549
Rp	2	11962253	3513203	2942767	0.8376	50647
Rp	3	8166359	2380137	1957370	0.8224	51329
Rp	4	8683279	1822029	1544658	0.8478	45396
Rp+V	1	12039463	2386758	2094851	0.8777	47374
Rp+V	2	12488290	3063769	2681615	0.8753	49295
Rp+V	3	11230683	2336137	2035010	0.8711	47213
Rp+V	4	15247311	2809497	2458482	0.8751	48850
Rp+M	1	12035705	2706510	2340246	0.8647	49335
Rp+M	2	14060261	3623641	3133965	0.8649	52420
Rp+M	3	9409195	3318129	2788195	0.8403	54893
Rp+M	4	14070202	3475382	3047851	0.877	49675
Rp+V+M	1	4762902	810063	742781	0.9169	45144
Rp+V+M	2	8278437	1786446	1640589	0.9184	44020
Rp+V+M	3	6359470	1413777	1264975	0.8947	46895
Rp+V+M	4	14212015	2929608	2582185	0.8814	48619

Dataset S1. Mutants with significant fitness differences identified from randomization tests, grouped according to function, metabolism, and direction of fitness differences.

Dataset S2. Normalized transposon insertion reads that are curated to the central 90% of coding region.

Dataset S3. Mean relative fitness (W) of mutants from four biological replicates. P-values were calculated for 10000 permutations of randomization tests, and were adjusted for multiple testing.
